# Regulation of cancer stem cells in triple negative breast cancer

**DOI:** 10.20517/cdr.2020.106

**Published:** 2021-06-19

**Authors:** Norman Fultang, Madhuparna Chakraborty, Bela Peethambaran

**Affiliations:** ^1^Department of Pathology and Laboratory Medicine, University of Pennsylvania, Philadelphia, PA 19140, USA.; ^2^Department of Biological Sciences, The University of the Sciences, Philadelphia, PA 19140, USA.

**Keywords:** Cancer stem cells, TNBC, transcriptional regulation, breast cancer

## Abstract

Triple Negative Breast Cancer (TNBC) is the most lethal subtype of breast cancer. Despite the successes of emerging targeted therapies, relapse, recurrence, and therapy failure rates in TNBC significantly outpace other subtypes of breast cancer. Mounting evidence suggests accumulation of therapy resistant Cancer Stem Cell (CSC) populations within TNBCs contributes to poor clinical outcomes. These CSCs are enriched in TNBC compared to non-TNBC breast cancers. The mechanisms underlying CSC accumulation have been well-characterized and discussed in other reviews. In this review, we focus on TNBC-specific mechanisms that allow the expansion and activity of self-renewing CSCs. We highlight cellular signaling pathways and transcription factors, specifically enriched in TNBC over non-TNBC breast cancer, contributing to stemness. We also analyze publicly available single-cell RNA-seq data from basal breast cancer tumors to highlight the potential of emerging bioinformatic approaches in identifying novel drivers of stemness in TNBC and other cancers.

## Introduction

Breast cancer remains the most commonly diagnosed cancer in women and the second leading cause of cancer-related deaths in women worldwide^[1]^. Breast cancer represents a highly heterogeneous group of neoplasms encompassing varied cell phenotypes with significant clinical implications. To aid characterization and guide therapeutic decisions, breast cancer has been stratified into several morphologically and molecularly distinct subtypes. These include Luminal A/B, Human Epidermal Growth Factor (HER2)-enriched, and Triple Negative (TNBC) subtypes^[2]^. TNBC is the most aggressive subtype of breast cancer and is characterized by hyperproliferative cells lacking expression of hormone receptors (estrogen and progesterone) and HER2^[3,4]^. Therapeutic avenues for TNBC are often limited due to the absence of these receptors and mortality rates far exceed other subtypes^[3,5-7]^. Additionally, TNBCs respond less to conventional chemotherapy and patients are at increased risk of recurrence and relapse^[5,7]^. Overall survival also lags far behind other non-TNBC subtypes predominantly due to therapy failure and/or relapse^[1,3,5,7]^. Accumulating evidence now suggests that TNBCs are enriched in therapy resistant Cancer Stem Cells (CSC), compared to non-TNBC subtypes, which significantly contribute to heightened mortality, therapy failure, and recurrence^[8-10]^.

CSCs are a small population of self-renewing tumor cells that persist after therapy and differentiate into all the cell types within the original tumor, reprising its heterogeneity^[11,12]^. Originally identified as self-renewing cells in leukemia with a CD34^+^CD38^-^ phenotype, they have since been identified in several solid and hematologic malignancies, including breast cancer, as both hyperproliferative and slow-cycling cells with a consensus CD44^+^CD24^-^/ALDH1^+^ phenotype^[11]^. These tumor-initiating CSCs are especially adept at repopulating tumors in animal disease models compared to non-CSC tumor cells^[12]^.

During embryonic development and throughout life, highly plastic stem cells differentiate into various cell and tissue types in processes spatiotemporally regulated by developmental pathways such as the wingless signaling pathway (Wnt)/β-catenin, Notch (neurogenic locus notch homolog protein 1), and Sonic Hedgehog pathways^[11,13-15]^. During carcinogenesis, aberrant regulation of these pathways allows cancer cells to acquire a “stem-cell”-like phenotype with increased ability to proliferate, tolerate hostile environments, and differentiate into different cell types^[13,14,16]^. This stem-cell oncogenic conversion is also mediated by factors within the tumor microenvironment (TME) and deregulated epigenetic and transcriptional programs^[16-18]^.

In TNBC, deregulation of stemness pathways is even more pronounced than in non-TNBC breast cancers, conferring TNBC CSCs (TNBCSCs) an especially problematic clinical phenotype^[8-10]^. Indeed, several analyses of human breast carcinomas have revealed TNBCs harbor the highest percentage of CD44^+^CD24^-^ALDH1^+^CSCs, a feature that usually correlates negatively with chemotherapy response, disease-free survival, metastasis-free survival, and overall survival^[19]^. In TNBC patients, chemotherapy and radiotherapy eradicate most hyperproliferative cells within the TME but fail to kill quiescent, slow-cycling TNBCSCs, allowing them to reinitiate the tumor (source). TNBCSCs are resistant to a host of therapeutic agents-a phenomenon referred to as Multi-Drug Resistance (MDR)^[20-22]^. The mechanisms of MDR, as well as new therapeutic approaches being pursued in both CSCs and TNBCSCs, have been reviewed extensively in previous publications^[20,23]^. This review focuses on cellular pathways and epigenetic and transcriptional factors that regulate stemness in TNBC. We also perform an analysis on publicly available TNBC single-cell RNA sequencing [single cell ribonucleic acid sequenceing (scRNA-seq)] data to highlight the potential of emerging systems biology approaches in identifying novel drivers of stemness in TNBC and other cancers.

## Cellular pathways regulating stemness in TNBC

**Figure 1:**
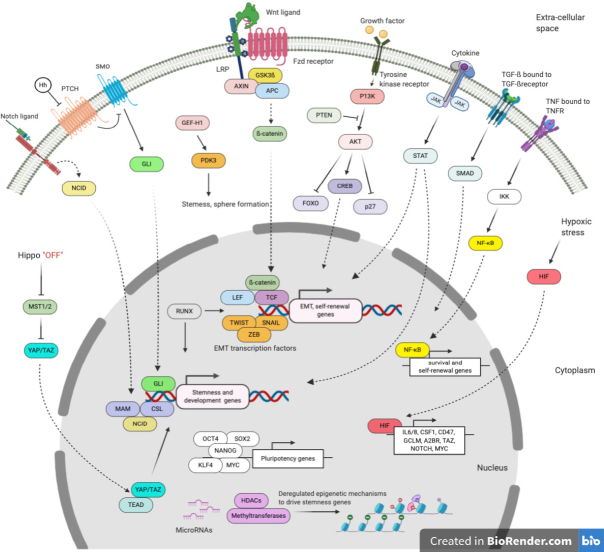
Signaling pathways and epigenetic and transcriptional mechanisms deregulated in TNBC contributing to stemness. Created using Biorender.com

Compared to other subtypes of breast cancer, TNBC cells are enriched in several stemness pathways. These include classical stem-cell pathways such as Wnt/β-catenin (beta), Notch, and Hh, which regulate cell fate and tissue patterning in early life, as well as cZEBell proliferation axes such as JAK/signal transducer and activator of transcription 3 (STAT3)/5^[11,13-15,24]^. Regulators of Epithelial-to-Mesenchymal Transition (EMT), an essential developmental process during organogenesis, including SNAIL, and twist family bHLH transcription factor 1 (TWIST) transcription factors, are also vital to TNBC stemness^[25-27]^. Recent evidence also suggests TME hypoxia and increased activity of pluripotency mediators such as OCT4, SOX2, and master regulator of cell cycle entry and proliferative metabolism (MYC), in TNBC compared to non-TNBC breast cancer, contribute to the elevated stemness phenotype^[28-30]^. These and several other molecular regulators of TNBC stemness are evaluated in this section [Figure 1].

### Wnt/β-catenin

The non-canonical and canonical Wnt signaling pathways are highly conserved developmental pathways governing cell polarity and tissue patterning in early life^[13]^. Canonical Wnt signaling is initiated by binding of Wnt ligands to either frizzled domain (FZD) or low-density-lipoprotein (LRP) family receptors resulting in the formation of a Wnt complex. This complex recruits and occupies members of the β-catenin destruction complex, preventing proteasomal degradation of β-catenin. Stabilized β-catenin then translocates to the nucleus where it acts as a transcriptional coactivator in combination with T-cell factor (TCF) and lymphoid enhancer-binding factor (LEF)^[13]^. Non-canonical Wnt signaling is also initiated by Wnt ligand binding but does not culminate in β-catenin stabilization. Both pathways result in increased proliferation, loss of E-cadherin/EMT, mammosphere formation, invasiveness, and colony formation^[13,17,31]^.

Aberrant activation of Wnt signaling is a hallmark of several cancers including breast cancer^[13,14,31,32]^. Compared to non-TNBC breast cancer, TNBC is enriched in a number of Wnt pathway genes^[32-34]^. Aberrant Wnt signaling has been linked with increased stemness and chemoresistance in TNBC and TNBCSCs. Xu *et al.*^[35]^ showed highly active Wnt-signaling is required for TNBC tumorigenesis, migration, stemness, anchorage-independent growth, and chemoresistance. In a 4T1-mouse model of TNBC, Jang *et al*.^[36]^ showed Wnt-signaling is enriched in TNBCSCs compared to the bulk tumor population. Activation of Wnt-signaling via WNT3A treatment significantly increased the number of ALDH+ TNBCSCs, while inhibition of Wnt-signaling had the opposite effect.

Wnt signaling was also shown to promote CSC cycling via transcription of cell cycle regulators Cyclin D1 and MYC^[37]^. Wnt signaling also contributes to dedifferentiation of breast cancer cells into pluripotent CSCs^[38,39]^; Wnt co-receptor LRP8 was similarly shown to promote TNBCSCs via conversion into a basal-mesenchymal stem-cell-like phenotype^[40]^. Non-canonical Wnt receptors including receptor tyrosine kinase like orphan receptor 1 (ROR1) and receptor tyrosine kinase like orphan receptor 2 (ROR2), which are especially enriched in ER negative breast cancer^[33,41]^, have been shown to promote stemness in ER negative breast cancer via interactions with Yes associated Protein 1 (YAP)/WW domain-containing transcription regulator protein 1 (TAZ) and BMI-1 stemness pathways^[42]^. Additionally, several non-Wnt molecules, including Enhancer of zeste homolog 2 (EZH2), telomerase reverse transcriptase (TERT), Capillary morphogenesis gene 2 (CMG2), Histone-lysine N-methyltransferase (SMYD3), CD138, and Focal adhesion kinase (FAK), regulate CSCs in TNBC and other cancers via their interaction with Wnt effectors such as β-catenin and LRP6^[43-48]^. Finally, Cleary *et al*.^[49]^ also demonstrated that a persistent population of tumor-initiating cells could reactivate Wnt-signaling following Wnt inhibition, repopulating the tumor and contributing to relapse. This finding underlines a core issue with targeting Wnt clinically-Wnt inhibitors usually abrogate Wnt signaling in tumor cells but not in CSCs, which persist and can rescue the hyperactive Wnt phenotype^[49]^. It is imperative that emerging therapeutic approaches target Wnt in CSCs. Altogether, the data from these studies suggest a vital role for Wnt signaling in TNBCSCs and TNBC recurrence.

### Notch signaling

Notch signaling is a highly conserved developmental pathway that is triggered when Notch ligands (Delta-like-1/3/4, Jagged1, and Jagged2) bind to one of several Notch receptors (NOTCH1-4) on neighboring cells, triggering the latter’s proteolytic cleavage and nuclear translocation^[50-52]^. In the nucleus, cleaved NOTCH binds transcription factors *C*BF-1/RBP-jκ, *S*u(H), *L*ag-1 (CSL) and Mastermind (MAM) to form a ternary NICD (*n*otch *i*ntracellular *d*omain)/CSL transcription complex that regulates the expression of target genes^[50,51]^.

Notch is aberrantly expressed in breast cancer CSCs where it promotes self-renewal and metastasis^[53,54]^. Notch is more significantly deregulated in TNBC compared to non-TNBC breast cancer-in fact, Notch ligands have been suggested as clinical markers for TNBC^[52,55]^. Increased Notch signaling in TNBCSCs might be mediated by transcription factor KLF4 and growth factor BMP4 Yu *et al*.^[56]^ and Choi *et al*.^[57]^ demonstrated that KLF4 and BMP4 increased Notch1 and Jagged1 expression in TNBCSCs, promoting cell motility and invasiveness. In another study, hypoxia, a hallmark of TNBC^[58]^, induced Jagged1 expression in TNBCSCs, promoting metastasis and self-renewal^[59]^. Cell polarity regulator NUMB, which negatively regulates Notch-signaling, was also recently shown to be downregulated in TNBC, contributing to increased EMT and stemness^[60]^. Furthermore, loss of F-box and WD repeat domain-containing protein 7 (Fwb7), a tumor suppressor significantly reduced in TNBC^[61]^, increases Notch activity in breast cancer^[62]^. Liubomirski *et al*.^[63]^ also recently highlighted a significant role for tumor-stroma interactions in promoting Notch activity in TNBC. Altogether, these studies highlight how Notch signaling is intricately regulated in TNBC to promote stemness and invasiveness.

### Hh signaling

Similar to Wnt and Notch, Hh signaling is an important developmental pathway co-opted by tumors to promote stemness and tumor persistence^[64,65]^. Hh signaling consists of Hh ligands binding to transmembrane receptor Protein patched homolog 1 (PTCH), regulating transmembrane protein smoothened (SMO), which induces downstream activation or repression of transcription via glioma-associated oncogene (GLI) proteins. SMO positively regulates Hh signaling while PTCH negatively regulates SMO. In the absence of Hh ligands, PTCH binds and inhibits SMO. In the presence of Hh ligands bound to PTCH’s extracellular domain, the latter undergoes a conformational change, preventing it from inhibiting SMO. SMO then induces downstream activation of GLI transcriptional regulators^[65,66]^. Individual GLI proteins, including Gli 1-3, have varying effects on the transcription of Hh target genes: *GLI1* is a transcriptional activator, *GLI3* a transcriptional repressor, and *GLI2* a dual context-dependent regulator^[64,65]^.

Normally, Hh signaling regulates morphogenesis in early life and proliferation in adult stem cells^[15]^. In TNBC, Hh signaling has been associated with highly proliferative high-grade disease, increased metastases, and worse disease-free survival^[67-69]^. Several transcriptional targets of Hh signaling in TNBC, including ABCB1, ABCG2, Forkhead box protein M1 (FOXM1), and BMI-1, confer TNBCSCs resistance to chemotherapy^[66,70]^. Hh signaling also induces several EMT and invasiveness regulators including Snail family zinc finger 1 (SNAI1), Neuropilin 2 (NRP2), Cysteine-rich angiogenic inducer 61 (CYR61), Matrix metallopeptidase (MMP), and C-X-C chemokine receptor type 4 (CXCR4)^[66]^. As with Notch signaling, tumor-stroma interactions have been shown to sustain Hh signaling in TNBC promoting stemness^[71]^.

Paradoxically, hypoxia-induced Carbonic anhydrase Carbonic Anhydrase (CAXII), which is highly expressed in TNBC, negatively regulates Hh signaling^[72]^. Similarly, pluripotency factor NANOG, which is equally overexpressed in TNBC^[73]^, was found to inhibit Hh-induced transcription^[74]^. These findings perhaps emphasize the temporal, intentional and context-dependent nature of Hh regulation in TNBC. Further work is needed to fully understand the spatiotemporal dynamics of Hh regulation within TNBCSCs and the TNBC microenvironment.

### Growth factor and cytokine-driven pathways

#### Guanine nucleotide exchange factor (GEF)-H1/PKD

The protein kinase D (PKD) family of actin remodeling proteins are well-characterized cell migration regulators in TNBC and other breast cancers^[75-77]^. PKD1 is the predominant isoform in non-malignant tissue where it maintains an epithelial phenotype. Upon oncogenic conversion, PKD1 is silenced via methylation inducing EMT^[78]^. Because they lack the expression of ER, a transcriptional repressor of PKD expression, TNBCs express high levels of PKD2 and PKD3^[79]^. PKD3, especially, is associated with increased TNBC metastasis, proliferation, and stemness^[78,80-82]^. Recently, Lieb *et al*.^[80]^ also demonstrated that upstream activation by Rho guanine nucleotide exchange factor 2 (GEF-H1) mediates PKD3 maintenance of TNBCSCs.

#### JAK/STAT

The JAK/STAT pathway is an evolutionarily conserved axis that plays a central role in several cellular processes including proliferation, motility, and stemness^[83]^. JAK/STAT signaling is initiated when a growth-factor or cytokine binds to a cell surface receptor containing a JAK binding site. JAK is recruited to the intracellular JAK binding site of the receptor where it is activated. Activated JAK then phosphorylates and activates a STAT transcription factor for downstream transactivation of target genes. During development, JAK/STAT is essential for several homeostatic processes including hematopoiesis, stem cell maintenance, and organogenesis^[83,84]^. It also plays a key role as a pluripotency mediator for somatic cell reprogramming^[85]^. Constitutive activation of JAK/STAT signaling has been well-characterized as a driving factor in several malignancies^[86]^. In breast cancer, JAK/STAT has been identified as a key regulator of CSC self-renewal and non-CSC cells’ dedifferentiation into CSCs^[87,88]^.

Certain growth factors and cytokines that activate JAK/STAT have been identified as essential drivers of TNBC proliferation and stemness. These include IL6, Prostaglandin-I synthase (PTGIS), Hyaluronan synthase 1 (HAS1), C-X-C Motif Chemokine Ligand 3 (CXCL3), and 6-phosphofructo-2-kinase/fructose-2, 6-biphosphatase 3 (PFKFB3)^[88]^. Additionally, the IL-6/JAK2/STAT3 pathway is preferentially activated in TNBCSCs compared to non-TNBC BC and is associated with increased risk of metastasis^[88]^. Other cytokines including IL6, IL8, and CXCL1, which similarly drive JAK/STAT, are associated with increased growth and stemness in TNBC but not non-TNBC breast cancer^[89,90]^. Leptin, an energy homeostasis regulator enriched in TNBC, drives JAK2/STAT3 activity TNBCSCs, promoting stemness^[91]^. HN1L, an upstream regulator of STAT3, is also enriched in TNBC where it is associated with poor clinical outcomes, stemness, and motility^[92]^. This is mediated by its regulation of STAT3, LEPR, and pluripotency regulators SOX2/9 and KLF4^[92]^. Other chemokines including CCL5 have been shown to promote spheroid formation in TNBC via JAK/STAT^[93]^. IFN-β, which is repressed in TNBC, was also shown to negatively regulate TNBCSC formation via activation of STAT1^[94]^. Intriguingly, in luminal breast cancers, IFN-β promotes stemness via induction of SOX2 and STAT3 activity^[95]^. In TNBC, IFN-β does not activate STAT3. This suggests IFN-β regulation of stemness, and by extension general CSC regulation, is subtype-specific and context-dependent.

#### Transforming growth factor beta and tumor necrosis factor

Transforming growth factor beta (TGF-β) and tumor necrosis factor (TNF) are two important, antagonistic cytokines, which regulate a plethora of cellular activities including differentiation, survival, proliferation, and homeostasis^[96]^. They have been implicated in the progression of several cancers^[97]^.

TGF-β is important for early mammary development, regulating morphogenesis via specific regulation of ECM remodeling, and epithelial cell growth and differentiation^[98]^. Its role in breast cancer, however, is a lot more complex. Early in breast cancer development, TGF-β inhibits cell growth and promotes apoptosis. In later stages, it promotes proliferation, invasiveness, and stemness^[99]^. TGF-β also promotes EMT in breast and other cancers via activation of downstream transcriptional effectors small mothers against decapentaplegic (SMAD), SNAIL, Zinc-finger E homeobox-binding family (ZEB), and TWIST^[100-103]^. Shipitsin *et al*.^[104]^ showed that TGF-β is preferentially expressed in ER- CSCs where it regulates differentiation into an epithelial phenotype. In TNBC, treatment with chemotherapeutic agent paclitaxel causes hyperactivation of autocrine TGF-β signaling, promoting therapy resistance and relapse^[105]^. TGF-β also directly regulates the expression of Wnt5a promoting stemness and proliferation^[106]^. TGF-β is often enriched in the TNBC microenvironment and can be produced by infiltrating stromal and immune cell populations^[107]^. These studies suggest TGF-β-induced CSC accumulation as a drug resistance mechanism in TNBC.

TNFα is an inflammatory cytokine secreted predominantly by activated macrophages, natural killer cells, MDSCs, and T-cells in the TME^[97,108]^. Although initially thought to be an effector for anti-tumor immunity, TNFα has been shown to have some pro-tumor functions. TNFα promotes plasticity, angiogenesis, and CSCs in breast cancer^[109]^. TNFα secreted by tumor-associated-macrophages has also been shown to induce EMT and stemness pathways via activation of NF-κB (Nuclear factor kappa-light-chain-enhancer of activated B cells)^[110]^. Liu *et al*.^[111]^ demonstrated that TNFα increases TNBCSCs via the upregulation of TAZ and NF-κB. These findings closely mirror works from Storci *et al*.^[112]^ and Li *et al*.^[113]^, who showed that TNFα increases CSCs and EMT in TNBC via upregulation of *SNAIL*-related zinc-finger transcription factor (SLUG) and TWIST. Altogether, these studies suggest a vital role for inflammatory cytokines secreted by tumor and infiltrating immune cells in regulating stemness and self-renewal in TNBC.

#### PI3K/protein kinase B/mechanistic target of rapamycin kinase(mTOR)

The PI3K/Protein Kinase B (AKT) pathway is a ubiquitous growth pathway that regulates cell proliferation, survival, motility, and differentiation in most tissue types^[114]^. PI3K/AKT hyperactivity has been associated with the progression of several cancers^[114]^. Recent studies have also linked aberrant PI3K/AKT activation to breast cancer stemness^[115,116]^. PI3K/AKT signaling results in phosphorylation and activation of AKT by mTOR and PDK1. Activated AKT regulates downstream effectors such as cAMP response element-binding protein (CREB), Forkhead box protein O1 (FOXO), p27, and mTOR, driving growth and motility. Phosphatase and tensin homolog (PTEN) directly antagonizes AKT activation regulating the pathway^[117]^. In TNBC, loss-of-function mutations to PTEN and gain-of-function mutations to PI3K drive an especially hyperactive PI3K/AKT phenotype^[118]^. This phenotype drives tumor heterogeneity and CSC accumulation via induction of a “fibroblast-like” state^[119]^. TNBCSCs also express higher levels of mTORC1 compared to the bulk of the tumor contributing to self-renewal and growth^[120]^. SRC Proto-Oncogene, Non-Receptor Tyrosine Kinase (SRC) kinase, which is an upstream activator of PI3K/AKT, induces TNBCSC enrichment and resistance to therapy^[121]^. HIF-2α drives TNBCSCs activation via induction of CD44 and PI3K/AKT/mTOR signaling^[115]^. Intriguingly, Britschgi *et al*.^[122]^ showed that inhibition of PI3K/AKT/mTOR in TNBC could inadvertently induce IL8 secretion and activate a JAK2/STAT5 axis which promotes stemness and metastases. This suggests compensatory signaling mechanisms might play a bigger role than previously thought in driving CSCs.

### EMT regulators

Phenotypic plasticity is the most characteristic feature of CSCs. These tumor-initiating cells reserve the ability to switch from a quiescent, epithelial state to a motile, drug-resistant mesenchymal phenotype capable of invading other tissues and re-seeding tumors^[109]^. EMT is thus a key feature of CSCs, and several EMT regulators, mostly transcription factors, play important roles in regulating CSC function. Several of these regulators, including downstream transcriptional effectors for Wnt (β-catenin/LEF/TCF), Notch (NCID/CSL), Hh (GLI), and TNF/TGF-β, are discussed above.

#### SNAIL

Wnt/β-catenin signaling induces SNAIL accumulation. SNAIL mediates E-cadherin repression, inducing EMT^[123]^. In TNBC, SNAIL is associated with relapse, chemoresistance, and metastases^[124]^. SNAIL also regulates IL-8 expression, which promotes stemness in TNBC^[125]^. A SNAIL-G9A-DNMT1 complex also epigenetically silences Fructose-Bisphosphatase 1 (FBP1) in TNBCSCs, inducing metabolic reprogramming increasing glucose uptake and ATP production even under hypoxia^[126]^. The SNAIL-induced glycolytic switch to a more “Warburg”-like state reduces oxygen consumption and reactive oxygen species (ROS) accumulation, promoting tumorigenesis, survival, and self-renewal^[126]^. Recent work by Zhang *et al*.^[25]^suggests uncoupling Protein 1 (UCP1), which is downregulated in TNBC, represses SNAIL-mediated FBP1 silencing, suppressing TNBCSC accumulation. BRD4 also promotes SNAIL expression in TNBC, conferring TNBC cells with stem-cell-like traits^[127]^.

#### ZEB

ZEB is another key transcriptional regulator of EMT and stemness. Similar to SNAIL and other EMT regulators, it represses E-cadherin and is induced by several stemness-associated signaling pathways including Wnt/β-catenin and cytokine signaling^[123]^. Pioneering work by Chaffer *et al*.^[26]^ suggests ZEB1 is a CSC-switch in TNBC. TNBC cells maintain the ZEB1 promoter in a bivalent chromatin configuration allowing them to quickly respond to microenvironmental cues, modulating ZEB1 expression to switch between non-CSC and CSC phenotypes. This challenges the current dogma of CSC activity that suggests CSCs give rise to non-CSC cells in a unidirectional manner. Instead, TNBCSCs and non-CSC TNBC cells reserve the ability to switch between stem cell and non-stem cell phenotypes depending on environmental stimuli. Intriguingly, this interconversion was not found in other non-TNBC breast cancer subtypes, suggesting ZEB1 modulation as a key mechanism for the problematic tumorigenicity of TNBCs. Feldker *et al*.^[128]^ also showed that ZEB1 forms a transactivation complex with AP-1 factors FOS like 1, AP-1 transcription factor subunit (FOSL1), JUN, and Hippo pathway effector YAP in TNBC cells, promoting stemness genes’ expression.

#### TWIST

TWIST is the third major transcriptional regulator of EMT. TWIST regulates stemness in TNBC by downregulating CD24, inducing the accumulation of CD44^+^CD24^-^ALDH^+^TNBCSCs^[27]^. Additionally, a TWIST/BRD4 complex induces IL31RA expression in TNBC cells, promoting stemness via IL31^[129]^. A similar TWIST/BRD4 complex transcribes WNT5A in TNBC, promoting stem-cell-like properties and tumorigenicity^[130]^. In TNBC, deubiquitinating enzyme ubiquitin-specific protease 2 (USP2) promotes TWIST stabilization, allowing it to induce stemness, EMT, and chemoresistance^[131]^. TWIST also increases the expression of drug efflux pumps in TNBCSCs, promoting chemoresistance^[27]^.

### Hypoxia

Hypoxia is a vital contributing factor to CSC generation and maintenance. Hypoxia is a hallmark of the TME in several cancers, and it drives CSC accumulation via the activity of Hypoxia-Inducible Factor (HIF) transcription factors^[30,132]^. HIFs normally function to maintain oxygen homeostasis, preventing excessive production of toxic ROS^[132]^. The TNBC TME is highly hypoxic^[58]^. Treatment with chemotherapeutic agents such as paclitaxel and gemcitabine exacerbates hypoxia in the TNBC TME, inducing HIF activity^[30,133]^. These HIFs promote TNBCSC accumulation via induction of IL-6 and IL-8 signaling and increased MDR1^[30]^. Additionally, HIFs regulate the production of Colony Stimulating Factor 1 (CSF1) in TNBCs, which recruits tumor-associated macrophages and myeloid-derived suppressor cells to the TME^[134]^. In the TNBC TME, these pro-tumor immune cell types secrete cytokines such as TGF-β and tumor necrosis factor (TNF-α), which drive stemness and metastases, as discussed above^[134]^. HIF-1 also transcribes CD47 in TNBCSCs; CD47 allows TNBCSCs to evade phagocytosis by macrophages^[135]^. HIF-1 also promotes the expression of Glutamate-Cysteine Ligase Modifier Subunit which inhibits mitogen-activated protein kinase kinase (MEK)/extracellular-signal-regulated kinase (ERK) signaling in TNBC cells. Loss of MEK/ERK signaling promotes nuclear translocation of FOXO3, which transcribes and activates TNBCSC and pluripotency mediator NANOG^[133]^. Similar findings by Lan *et al*.^[136]^ suggest HIF-1-induced A2BR activates Protein kinase C delta type (PKC-δ)/STAT3 to transcribe IL-6 and NANOG promoting stemness in TNBC. HIF-1 also directly transcribes TAZ, which induces TNBCSCs^[137]^. Work by Lee *et al*.^[29]^ also suggests amplified MYC and MCL1 in TNBC drive the production of ROS, which in turn induce HIF-1α, promoting stemness and chemoresistance.

Carbonic anhydrase CAIX is another hypoxia-induced factor directly regulated by HIF allowing cancer cells to regulate intracellular pH during hypoxia^[138]^. It is highly expressed in TNBC where it correlates with poor survival and metastases as well as promotes TNBCSC survival and stemness^[138-140]^.

## Other transcription regulatons of TNBC stemness

### Pluripotency regulators

Pluripotency is a common feature of both CSCs and normal stem cells. During development, pluripotency is maintained and induced by a group of transcription factors regulated by environmental clues to create varied cell and tissue lineages^[141]^. Ectopic overexpression of these transcription factors, including OCT4, SOX2, NANOG, KLF4, and MYC, on non-malignant somatic cells can reprogram them into pluripotent stem cells^[141,142]^. Perhaps unsurprisingly, TNBC and other tumors similarly overexpress these factors to promote pluripotency and self-renewal.

OCT4 was first identified as an essential regulator of pluripotency and self-renewal in the embryo during development^[143]^. It has since been identified as a significant oncogene promoting stemness, self-renewal, and tumor recurrence^[144]^. OCT4 is overexpressed in TNBC where it correlates with worse clinical outcomes^[28]^. Cheng *et al*.^[145]^ showed that STAT3 drives OCT4 and MYC expression in TNBCSCs increasing chemoresistance and TNBCSC accumulation. Thiagarajan *et al*.^[91]^ further demonstrated that leptin induced STAT3 promotion of OCT4 expression was specific to TNBCSCs and not non-CSC TNBC cells. Lu *et al*.^[133]^ also showed that chemotherapy induces a HIF/S100A10/KDM6A axis which promotes OCT4 expression and stemness in TNBC. Interestingly, emerging work from Jin *et al*.^[146]^ recently suggests OCT4 can suppress EMT in both TNBC and Luminal cancers via activation of STAT3. This suggests potential bimodal regulation of stemness and self-renewal by OCT4.

SOX2 is another regulator of stem cell pluripotency during embryonic development^[141]^. SOX2 is overexpressed in TNBC where it correlates with increased proliferation, metastasis, and worse clinical outcomes^[147,148]^. Mukherjee *et al*.^[149]^ found that SOX2 was increased in TNBCSCs induced by chemotherapy, promoting resistance and survival. Jung *et al*.^[150]^ showed that, in TNBC, SOX2 activity is predominantly localized to TNBCSCs. SOX2 upregulation in TNBCSCs might be mediated by an IMP3/SLUG signaling axis which transcribes SOX2 specifically in TNBC^[151]^. VEGF, a potent angiogenic factor in the TME, also drives TNBCSC accumulation and activity by promoting VEGF/STAT3 transcription of SOX2 and MYC^[152]^. LIPH was also shown to regulate SOX2 in TNBCSCs, promoting metastasis^[153]^.

NANOG is a master regulator of self-renewal and pluripotency highly expressed in early life but silenced in adult somatic cells^[154]^. In several cancers, aberrant reactivation of NANOG contributes to tumorigenicity and stemness^[141]^. NANOG has been linked with increased stemness and poorer clinical outcomes in TNBC^[155,156]^. Paradoxically, in a follow-up study, Nagata *et al*.^[73]^ found NANOG to be a favorable prognostic marker for TNBC. This suggests NANOG might not be a reliable biomarker for TNBC. It is, however, a key driver of TNBC stemness, self-renewal, and metastasis^[155,157]^. NANOG overexpression in TNBC is driven by p38γ MAPK activation, resulting in c-JUN/AP-1 transcription of the *NANOG* gene^[157]^. Thiagarajan *et al*.^[155]^ also showed that Cx26 forms a signaling complex with NANOG and FAK, stabilizing NANOG driving stemness. The formation of this complex was specific to TNBC and not present in non-TNBC cells. As mentioned above, HIF-1 activity also directly induces NANOG transcription in TNBCSCs via MEK/ERK/FOXO3 and PKC-δ/STAT3^[133,136]^.

KLF4 is another important transcription factor during development. In tumors, it has been shown to have both oncogenic and anti-cancer roles^[158]^. In TNBC, its role appears to be equally confounding. Nagata *et al*.^[73,159]^ showed it is predominantly tumor-suppressive and associated with favorable clinical outcomes in TNBC by suppressing EMT. However, Yu *et al*.^[56]^ showed it is essential for the maintenance of TNBCSCs and TNBC migration and invasion. Zhou *et al*.^[160]^ also showed that KLF4 accumulation in TNBC is sustained by Protein arginine methyltransferase 5 (PRMT5) -mediated methylation, which prevents KLF4 degradation. Accumulation of KLF4 promotes TNBCSC expansion and survival^[160]^. Sharma *et al*.^[161]^ similarly demonstrated that KLF4 is predominantly expressed in TNBCSCs where it promotes survival, self-renewal, and chemoresistance. They also showed receptor tyrosine kinase (RTK) is a transcriptional target of KLF4 in TNBC, partially contributing to the chemoresistance phenotype^[161]^.

The MYC family of pro-oncogenic transcription factors are ubiquitous gene regulators that regulate several cellular processes including motility, survival, stemness, therapy resistance, and differentiation^[162]^. In several cancers, they are constitutively activated, driving hyperproliferative phenotypes^[162]^. In TNBC, MYC is highly expressed in CSCs where it drives self-renewal and chemoresistance^[29,163,164]^. Yin *et al*.^[164]^ showed that MYC drives accumulation of TNBCSCs and induction of EMT in TNBC. Lee *et al*.^[29]^ showed that MYC, in concert with MCL1, promotes mitochondrial oxidative phosphorylation, which induces HIF1 to promote TNBCSC accumulation and chemoresistance. In TNBC, high MYC levels are driven by a vascular endothelial growth factor receptor(VEGFR) -2/STAT3 axis, which transcribes MYC and SOX2 to promote stemness^[152]^. Lee *et al*.^[165]^ also showed that Hsp90α promotes nuclear translocation of c-MYC to transcribe CSC-mediator BMI-1 in TNBCSCs. ID proteins were also shown to activate MYC in TNBCSCs via negative regulation of Roundabout homolog 1 (ROBO1)^[166]^. As mentioned above, WNT signaling also promotes MYC and Cyclin D1 expression in CSCs driving self-renewal^[37]^.

### Runt-related transcription factor

The Runt-related transcription factor (RUNX) family of transcription factors regulate a plethora of developmental processes including cell growth, differentiation, and lineage specification^[167]^. During mammary development, RUNX factors are important for the maintenance of mammary epithelium homeostasis^[168]^. Work from Fritz *et al*.^[169]^ suggests RUNX factors drive EMT and stemness in breast cancer CSCs. In TNBC, RUNX1 is an independent prognostic indicator of poor patient outcomes^[170]^. In TNBCSCs, RUNX transcription factors and their coregulator CBFβ promote phenotypic plasticity and are essential for maintaining of the mesenchymal, invasive phenotype^[171]^. RUNX1 was also recently shown to regulate R-Spondin 3 (RSPO3) in TNBCSCs, promoting EMT, motility and stemness^[172]^. Accumulating evidence also suggests significant interaction between RUNX factors and the hippo pathway, an established self-renewal signaling axis in TNBC^[173]^.

### Hippo YAP/TAZ

The Hippo pathway is a key regulatory axis for cell fate, organ development, tissue regeneration, and self-renewal during development^[174]^. It consists of a cytoplasmic kinase module composed of mammalian Ste20-like kinases (MST1/2), which, in response to environmental cues, phosphorylates and activates LATS1/2 kinases. Activated LATS1/2 phosphorylates and inactivates an oncogenic transcriptional module consisting of YAP, TAZ, and TEA Domain family member (TEAD), promoting their retention and subsequent degradation in the cytoplasm. When the hippo kinase module is “off”, YAP/TAZ translocates to the nucleus and induces transcription of TEAD target genes^[23,174]^.

Aberrations in Hippo signaling have been shown to induce dedifferentiation of mature cells into progenitor cells^[175]^. It has also been associated with induction of CSC accumulation in various cancers. In breast cancers, TAZ confers CSCs self-renewal and tumor-initiation capacities^[176]^. In TNBC cells, YAP has been shown to regulate the transcription of stem cell signature genes, promoting tumorsphere formation^[177]^. Guo *et al*.^[178]^ demonstrated that YAP promotes chemoresistance in TNBC. Aberrant regulation of YAP/TAZ in TNBC is mediated by the activity of an SRF-IL6 axis and glucocorticoid receptor signaling^[177,179]^. KIBRA, a tumor suppressor, also inhibits YAP/TAZ activation but is silenced in TNBC by chromosomal mutation^[180]^. USP1 also promotes TAZ stability by deubiquitination in TNBC, promoting proliferation and metastasis^[181]^. YAP/TAZ signaling is also induced by several pathways discussed in previous sections: ROR1/2 activate YAP signaling; ZEB complexes with YAP to drive stemness; and HIF and TNF induce TAZ.

### NF-κB

The NF-κB transcriptional complex is a highly conserved transcriptional complex that regulates cell survival, growth, differentiation, and cytokine production^[182]^. It regulates gene expression in response to a plethora of extracellular stimuli including cytokines, free radicals, and pathologic antigens, among others^[182]^. The NF-κB family consists of five transcription factors: RelA, RelB, c-Rel, NF-κB1, and NF-κB2. Deregulated NF-κB activity has been associated with tumorigenesis in several cancers^[182]^.

In breast cancer, TNBCs exhibit the highest levels of constitutively activated NF-κB^[183]^. Activated NF-κB drives accumulation of TNBCSCs via induction of JAG1/NOTCH signaling^[183]^. This mechanism of CSC accumulation was found to be exclusive to TNBCs and not other breast cancers. Hossain *et al*.^[184]^ also showed that JAG1/NOTCH signaling in turn promotes NF-κB activity in TNBCSCs to transcribe anti-apoptosis gene cIAP-2. These findings suggest a dynamic, cyclical, NF-κB-JAG1/NOTCH-NF-κB signaling axis maintains TNBCSCs accumulation and survival in TNBCs. Intriguingly, the NF-κB inhibitor IKKε was also suggested as a key mediator of TNBCSC accumulation^[185]^. In concordance with these findings, Kim *et al.*^[87,177]^ and Barbie *et al*.^[93]^, showed that IKKε expression marks a population in TNBCs with high IL-6, a cytokine previously shown to promote stemness.

## Identifying new TNBCSC regulators

Significant leaps have been made in recent years developing bioinformatic tools to analyze and characterize heterogeneous tissue types. One such emerging technique, single cell RNA-sequencing (scRNA-seq), allows investigators to probe gene expression at the single cell level, identifying novel cell types and conserved biomarkers that drive their phenotype. ScRNA-seq has been used to identify novel, self-renewing CSC populations in several cancers^[186-188]^. We sought to similarly identify CSC populations within publicly available breast cancer scRNA-seq data, to demonstrate how high throughput scRNA-seq data can be used to identify new TNBCSC regulators.

scRNA-seq data from Yeo *et al*.^[189]^ profile the transcriptomes of over 13,000 cells from multiple mice models of breast cancer including TNBC(NCBI Geo accession number: GSE123366). Detailed methods on how the samples were prepared and data acquired can be found in^[189]^. Briefly, following tumor excision and dissociation into single-cell suspensions, breast epithelial cells were sorted via FACS and sequenced with the Chromium 10X droplet-based scRNA-seq platform. Library generation was performed using the 10X Chromium single-cell kit and pooled libraries sequenced using the Illumina HiSeq 2500 platform. Raw sequencing data were processed and normalized using the Cell Ranger Single Cell Software Suite. Cells with high mitochondrial gene ratio (> 20%) and low/high gene expression (< 500 genes; > 1000 genes) were excluded from the analysis.

**Figure 2:**
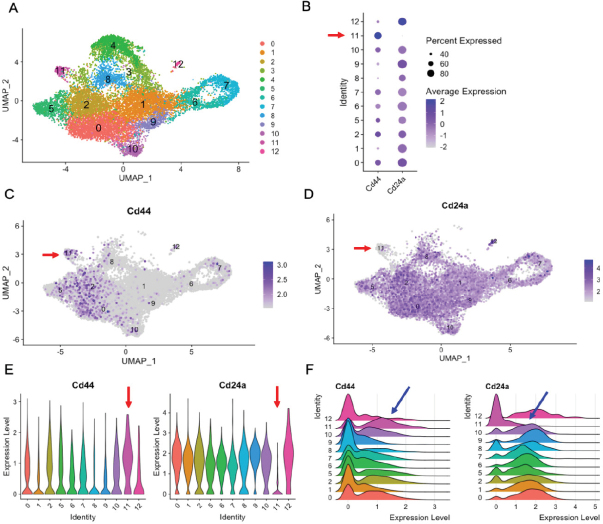
Red and blue arrows indicate putative CD44^-high^/CD24^-low^ CSC cluster (Cluster 11). (A) Combined Seurat analysis of 13,412 cells shown in UMAP projection plot showing the individual clusters identified in GSE123366; (B) dot plot visualizing CD44 and CD24 expression in the individual clusters; (C, D) feature plots showing CD44 and CD24 expression in individual cells; and (E, F) violin and ridge plots showing distribution of CD44 and CD24 expression in Seurat clusters, respectively

We analyzed the raw scRNA-seq matrix file deposited by the authors on NCBI GEO (GSE123366) using the Seurat package in R^[190]^. Data were fed into R as a counts matrix file, scaled by a factor of 10,000, and normalized by log transform. We further trimmed the data excluding cells with a mitochondrial ratio > 5% and included cells with more than 200 but fewer than 6000 genes expressed. In total, 13,412 cells were recovered after our trimming. Using Seurat’s *IntegrateData* function, the cells from all conditions were integrated into one analysis. Clustering was performed with the run principal component analysis in seurat (RunPCA) function using the first 20 principal components and visualized using uniform manifold approximation and projection (UMAP). Twelve distinct clusters were identified [Figure 2A]. To identify CSC populations, we profiled the expression of CD44 (Cd44) and CD24 (Cd24a) using dot, feature, violin, and ridge plots [Figure 2B-F]. We identified one small cluster (Cluster 11) as the only CD44^+^CD24^-^ cluster. Using the *FindAllMarkers* function, we identified genes specifically expressed/upregulated in Cluster 11 compared to other cells in the analysis [Table 1].

**Table 1:** Top 30 genes upregulated in CD44^-high^/CD24^-low^ cluster (Cluster 11). The genes were obtained using the Finallmarkers function from the Seurat package with expression threshold set at 0.25.

Gene	*P* value
Pcdh7	1.09E-150
Dcn	5.55E-119
Plpp3	3.19E-106
Creb3l1	3.22E-104
Hexa	7.26E-94
Ppic	4.18E-87
Serpina3n	2.91E-85
Mmp14	9.55E-80
Snhg18	1.01E-75
Vcan	1.97E-75
Ifitm2	3.08E-74
Col4a1	1.59E-73
Gstm2	8.51E-65
Serf2	5.42E-60
Calu	1.40E-54
Marcks	2.86E-51
Bmp1	7.37E-50
Selenof	2.12E-49
Itm2c	1.42E-41
Rrbp1	2.50E-40
Vasn	3.81E-38
Col27a1	1.73E-37
Ugdh	2.18E-37
Il6st	1.15E-31
Ckap4	3.62E-30
Vat1	4.21E-30
Lamc1	9.67E-30
Pdia3	6.54E-26
Cyth3	8.66E-19

Pcdh7: Protocadherin-7; Dcn: decorin; Plpp3: phospholipid phosphatase 3; Creb3l1: CAMP responsive element binding protein 3 like 1; Hexa: hexosaminidase A; Ppic: peptidylprolyl isomerase C; Serpina3n: serine protease inhibitor A3N precursor; Mmp14-matrix metallopeptidase 14; Snhg18: small nucleolar RNA host Gene 18; Vcan: versican; Ifitm2: interferon induced transmembrane protein 2; Col4a1: collagen alpha-1(IV); Gstm2: glutathione S-transferase mu 2; Serf2: small EDRK-rich factor 2; Calu: calumenin; Marcks: myristoylated alanine rich protein kinase C substrate; Bmp-1 Bone morphogenetic protein-1; Selenof: selenoprotein F precursor; Itm2c: integral membrane protein 2C; Rrbp1: ribosome-binding protein 1; Vasn: vasorin; col27a1: collagen alpha-1 (XXVII); Ugdh: UDP-glucose dehydrogenase; Il6st: interleukin 6 signal transducer; Ckap4: cytoskeleton associated protein 4; Vat1: vesicle amine transport 1; Lamc1: laminin subunit gamma-1; Pdia3: protein disulfide-isomerase A3; Cyth3: cytohesin-3

Several genes identified as Cluster 11 markers have been described as CSC regulators in TNBC and other cancers. PCDH7 was shown to promote stemness and brain metastasis in TNBCSCs^[191]^. Array data from da Silveira *et al*.^[192]^ suggest transcription factor CREB3L1 is upregulated in breast cancer CSCs compared to the bulk of the tumor. MMP14 expression was shown to be double in TNBCSCs compared to the bulk of the tumor population^[193]^. Kulesza *et al*.^[194]^ found SERPINA3 to be overexpressed in melanoma CSCs where it promotes metastasis and invasion. Oktem *et al*.^[195]^ showed that VCAN is increased in prostate CSCs, especially when maintained as spheroids. MARCKS1, induced by LncZi2, was shown to promote self-renewal and accumulation of liver CSCs^[196]^. In glioma CSCs, VASN was found to be a critical link between hypoxia and Notch signaling to maintain CSCs^[197]^. UDPH has also been linked to prostate cancer stemness^[198]^.

Several of the markers we identified in our analysis have not been described within the context of CSCs or TNBCSCs. Transcription factors such as CREB3L1 and SERPINA3 are especially attractive as potential TNBCSC regulators because they could directly regulate the expression of known stemness genes. Further work investigating the chromatin binding activity of these factors to determine their target genes in TNBCSCs is necessary to characterize their role in stemness.

## Epigenetic regulators of stemness in TNBC

The epigenome is a crucial component of cell identity. Precise regulation of the epigenome in early development and throughout life is essential for determining cell fate, tissue patterning, and organogenesis. Epigenetic mechanisms including DNA methylation, chromatin modifications, and RNA interference have all been shown to play a role in regulating cell growth and pluripotency^[18,199,200]^. Deregulations in these mechanisms, especially at loci of growth and pluripotency related genes, have been associated with tumorigenesis and stemness in several cancers^[18,199-201]^. In TNBC, aberrations in epigenetic mechanisms are key contributing factors to the highly heterogeneous and stem cell-like phenotype often seen in patients^[199,202]^.

DNA methylation is significantly deregulated in most breast cancers including TNBC^[200,203]^. A comprehensive analysis of the TNBC methylome by Stirzaker *et al*.^[203]^ showed that hypermethylation in TNBC correlates with worse patient outcomes. Kagara *et al*.^[201]^ demonstrated that, in TNBC, hypomethylation of the promoter regions for CSC markers CD44, CD133, and Musashi-1 drove increased expression, contributing to stemness. Hypomethylation of these genes was specific to TNBC and correlated with a clinically aggressive phenotype^[201]^. Arginine methyltransferase PRMT1 was also shown to promote TNBC stemness, potentially via activation of STAT3^[204]^. Methylation of the gene for scaffold protein DAB2IP was also shown to be essential for TNBCSCs accumulation. Treatment with DNA methylation inhibitor decitabine reversed the hypermethylation phenotype, rescuing DAB2IP expression and reducing stemness^[205]^. Bao *et al*.^[206]^ also showed that TET1 and 5hmC are essential for H_2_O_2_-dependent self-renewal and accumulation of TNBCSCs.

Deregulated chromatin architecture is a feature of several cancers^[207]^. Histone modifications at loci of pluripotency and stemness genes can promote stem cell accumulation in TNBC^[200]^. Li *et al*.^[200]^ showed that histone methylation profiles for TNBCSCs differ starkly from non-CSC TNBC cells. H3K4me2 and H3K27me3 methylation of genes in stemness pathways Wnt and GnRH differed significantly in TNBCSC, potentially driving deregulated pathway activity^[200]^. Histone methyltransferase EZH2 was shown to maintain stemness and metastasis in TNBCSCs. Inhibition of EZH2 differentiated TNBCSCs into a luminal-like phenotype which was more sensitive to endocrine therapy^[208]^. Histone deacetylases (HDAC) HDAC1 and HDAC7 have also been shown to be specifically overexpressed in TNBCSCs compared to non-CSC tumor cells^[209]^. Caslini *et al*.^[210]^ later showed that HDAC7 binds and regulates transcription start sites for CSC genes including c-MYC, CD44, SLUG, and SMAD3. In studies by both Witt *et al*.^[209]^ and Caslini *et al*.^[210]^, HDAC inhibition with clinically available HDAC inhibitors could be used to target CSCs. Su *et al*.^[211]^ similarly showed that HDAC inhibitors could reprogram TNBCSCs into a less aggressive phenotype. Recent work from Darvin *et al*.^[212]^ suggests increased expression of HDACs in CSCs drives EMT-induced PD-L1 expression in BT-549 TNBCSCs. They also found reduced distribution of hypermethylated, repressive histones H3K9me3 and H3K27me3 at the promoter for PD-L1, partially contributing to high PD-L1 expression in CSCs^[212]^. Torres *et al*.^[213]^ also found that linker histone H1.0 regulates the differentiation state of cells within several tumors including TNBC with H1.0-low cells being pluripotent and stem-cell-like.

Deregulation in mechanisms of RNA interference, predominantly microRNAs (miRNA), can similarly contribute to CSC accumulation in TNBC. Li *et al*.^[214]^ identified a six-miRNA gene signature that regulates TNBCSC response to chemotherapeutic stress. Chemotherapeutic stress drove increased stemness and self-renewal in TNBCSCs, partially via modulation of these miRNAs, including miR-193a-5p, miR-92a-3p, miR-192-5p, miR-375, miR-155-5p, and miR-21-3p. MiR-34a was also shown to repress stemness in TNBC by targeting IMP3, a ribonucleoprotein highly expressed in breast cancer and TNBC CSCs^[215]^. Sun *et al*.^[216]^ showed that miR-223 is downregulated in TNBCSCs compared to non-CSC cells and functioned as a negative regulator of TNBCSC survival. MiR-203 is a well-characterized regulator of stemness and EMT in TNBC. It suppresses EMT, colony formation, and proliferation by targeting TP63^[217]^. Subsequent work suggests miR-203 is epigenetically silenced in TNBC, specifically via DNA methylation^[218]^. Wellner *et al*.^[219]^ further demonstrated that miR-203 is one of several miRNAs repressed by stemness transcription factor ZEB1 to promote CSC self-renewal. MiR-205 is another negative regulator of stemness in TNBC which targets ZEB1/2 and is repressed by Notch signaling^[220,221]^. Dong *et al*.^[222]^ demonstrated miR-139 suppresses tumorigenicity in TNBC by targeting CSC factor SOX8. MiR-200c also inhibits CSC-factor ZEB2 in TNBC, repressing EMT^[223]^. Altogether, these findings suggest that miRNAs that target known CSC regulators are likely to be negative regulators of TNBCSCs and could be investigated as biomarkers and therapeutics for TNBCSCs.

## Conclusion

In this review, we provide an exhaustive summary of known regulatory mechanisms for CSCs in TNBC. We highlight transcriptional, epigenetic, and growth factor mechanisms that contribute to stemness with an emphasis on TNBC-specific mechanisms. It is worth noting that other mechanisms identified as contributors to CSC biology in other non-TNBC cancers, not highlighted here, would similarly play a role in TNBCSCs. These TNBC-specific mechanisms, however, are appealing therapeutic targets to combat relapse, recurrence, and therapy failure in this especially hard-to-treat cancer. Current approaches being pursued to combat CSCs in TNBC have recently been reviewed elsewhere^[23,224]^. Our scRNA-seq analysis also suggests there are several potential drivers of CSCs in TNBC yet to be described. Further work identifying and characterizing these CSC regulators is imperative.

## Declarations

### Availability of data and materials

The scRNA-seq data analyzed in this review is available at: https://www.ncbi.nlm.nih.gov/geo/query/acc.cgi?acc=GSE123366

All/R code used to analyze the data is available upon request.

### Copyright

© The Author(s) 2021.
